# Design and Pilot Implementation of an Electronic Health Record-Based System to Automatically Refer Cancer Patients to Tobacco Use Treatment

**DOI:** 10.3390/ijerph17114054

**Published:** 2020-06-06

**Authors:** Thulasee Jose, Joshua W. Ohde, J. Taylor Hays, Michael V. Burke, David O. Warner

**Affiliations:** 1 Department of Anesthesiology and Perioperative Medicine, Mayo Clinic, Rochester, MN 55902, USA; warner.david@mayo.edu; 2 Center for Clinical and Translational Science, Mayo Clinic, Rochester, MN 55902, USA; ohde.joshua@mayo.edu; 3 Department of Medicine, Nicotine Dependence Center, Mayo Clinic, Rochester, MN 55902, USA; hays.taylor@mayo.edu (J.T.H.); burke.michael1@mayo.edu (M.V.B.)

**Keywords:** electronic health record, tobacco, smoking, cancer, opt-out

## Abstract

Continued tobacco use after cancer diagnosis is detrimental to treatment and survivorship. The current reach of evidence-based tobacco treatments in cancer patients is low. As a part of the National Cancer Institute Cancer Center Cessation Initiative, the Mayo Clinic Cancer Center designed an electronic health record (EHR, Epic^©^)-based process to automatically refer ambulatory oncology patients to tobacco use treatment, regardless of intent to cease tobacco use(“opt out”). The referral and patient scheduling, accomplished through a best practice advisory (BPA) directed to staff who room patients, does not require a co-signature from clinicians. This process was piloted for a six-week period starting in July of 2019 at the Division of Medical Oncology, Mayo Clinic, Rochester, MN. All oncology patients who were tobacco users were referred for tobacco treatment by the rooming staff (*n* = 210). Of these, 150 (71%) had a tobacco treatment appointment scheduled, and 25 (17%) completed their appointment. We conclude that an EHR-based “opt-out” approach to refer patients to tobacco dependence treatment that does not require active involvement by clinicians is feasible within the oncology clinical practice. Further work is needed to increase the proportion of scheduled patients who attend their appointments.

## 1. Introduction

Tobacco use is the leading cause of cancer morbidity and mortality in the United States, accounting for approximately 30% of all cancer mortality [1]. Tobacco use treatment of cancer patients is efficacious [2,3] but the reach of tobacco cessation treatment services among cancer patients is low [4]. Few cancer centers across the US consistently provide tobacco treatment services [5,6], and until recently approximately two-thirds of the National Cancer Institute (NCI)-designated comprehensive cancer centers did not provide even basic elements of tobacco treatment such as patient education brochures [5]. Even for those centers that provide access to tobacco treatment services, few patients access these services [7]. Continued tobacco use after cancer diagnosis increases the risk of treatment-related toxicities, cancer recurrence, morbidity, mortality and increases overall treatment costs [8,9,10,11,12]. Thus, new approaches are needed to address tobacco use as a clinical practice priority as well as to provide consistent access to tobacco treatment services. As part of the NCI Cancer Moonshot℠ program, the NCI launched the Cancer Center Cessation Initiative (C3I) to implement sustainable tobacco control initiatives within cancer centers across the US [13].

In 2018, the Mayo Clinic Cancer Center (MCCC) joined the C3I initiative. The MCCC implementation initiative is intended to refer all cancer patients who use tobacco to tobacco treatment specialists (TTS) at the Mayo Clinic Nicotine Dependence Center (NDC) [14]. The referral process was designed to include all patients regardless of their readiness to stop using tobacco and required patients unwilling to participate to actively “opt-out” of treatment by declining the NDC referral. The “opt-out” approach [15] offers promise to increase the reach of tobacco treatment services in clinical settings rather than referring only those patients who expressed an explicit desire for treatment (“opt-in”). For patients not yet ready to stop, an NDC referral can also add value by providing education about the impact of tobacco use to cancer treatment, and planning support and strategies for future stopping attempts.

A unique component of the implementation is to deliver a best practice advisory (BPA) alert within the electronic health record (EHR) system Epic^©^ to the staff who room the patient. These staff schedule the NDC appointment without requiring permission of the oncology provider (i.e., physician or advanced practice nurse and physician assistant), easing their workload. In this report, we describe the design and implementation of the BPA, and the results of pilot implementation of the new clinical workflow in an ambulatory oncology practice.

## 2. Materials and Methods 

**Setting:** The MCCC is a NCI-designated comprehensive cancer center with primary campuses in Minnesota, Florida, and Arizona, as well as a network of 12 community oncology practices across Minnesota, Wisconsin, and Iowa. The clinical practice has been supported by EHR vendor Epic^©^ Systems Corporations since 2018. Approximately 157,000 oncology patients receive care annually. The design and pilot implementation work described in this report was performed in the Division of Medical Oncology practice at Mayo Clinic in Rochester, MN, as a precursor to later implementation of the system across the MCCC.

**Ethical Approval**: This project was considered a Quality Improvement initiative under Mayo Clinic Policy and was thus exempt from Institutional Review Board review. It was conducted in accordance with 45 CFR 46.102.

**Design Process:** Initial interviews, including practice leadership and management stakeholders, and the observations of current practices were performed by study personnel to define the then current workflow related to tobacco use. The reporting and registry capabilities of Epic^©^ were also utilized to determine the then current tobacco use documentation and referral patterns. Major findings that informed the new workflow design included the following:

1. In Epic^©^, tobacco use status is recorded in the social history section. Although there is an existing policy mandating at least annual ascertainment of tobacco use status, this section is not consistently documented nor tracked for compliance.
2. Oncology providers did not consistently document conversations with their patients regarding tobacco use, and the diagnosis of “nicotine dependence” was often not included in the patients’ problem lists.
3. Oncology providers supported routine patient referral for tobacco use treatment, and were willing to encourage their patients to attend NDC appointments, but were wary of further electronic reminders or ordering systems that would add to their already considerable burden of EHR interactions.
4. Allied health staff who “room” the patients in the practice (i.e., bring the patient to the examination room and gather basic demographic and health information, designated hereafter as “rooming staff”) were interested in participating in a process that would help cancer patients address their tobacco use, even if it added to their responsibilities.

Based on these findings and the considerations described in the introduction, the following design principles were formulated:

1. Current tobacco use status should be routinely ascertained as part of the oncology patient rooming process.
2. All current (defined as any tobacco use in the past 30 days) tobacco users should be referred to the NDC regardless of the immediate intent to stop smoking using the “opt-out” paradigm; i.e., tobacco treatment referral is the default.
3. The referral process should involve minimal provider burden, such as additional reminders or co-signing of orders.
4. Clinical providers should be made aware of their patients’ current tobacco use and that they have been referred for tobacco use treatment so that they can encourage attendance.

**Figure 1:**
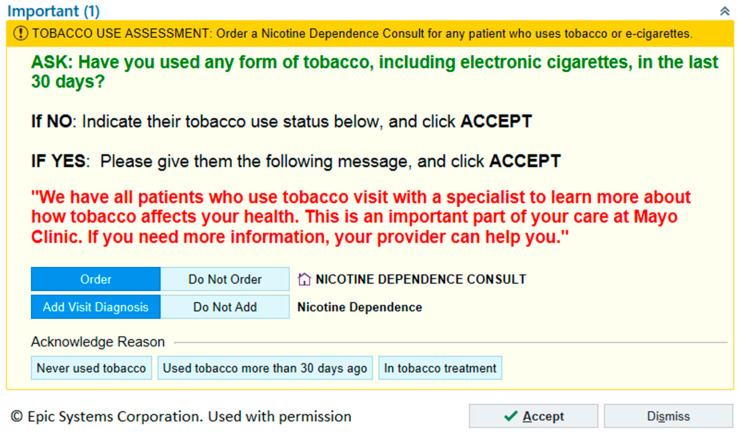
Epic^©^ best practice advisory alert for tobacco use intervention.

**BPA Design:** Based on these design principles, a BPA was created as a decision support tool in Epic^©^ (Figure 1). The BPA facilitates three tasks by the rooming staff. First, the staff asks about past 30-day tobacco use (including electronic cigarettes). For those who answer affirmatively, two additional tasks are accomplished: placing a request for NDC referral and adding “nicotine dependence” to the “visit diagnosis” list in the documentation of the subsequent provider encounter. These actions are set as the default choices of the BPA. A suggested script is provided for the rooming staff to utilize in informing the patient about the referral to the NDC. Patients are not provided with an option to decline the referral at this point; rooming staff are asked to direct all questions from the patients about the referral to their respective clinician. A clinician co-sign of the NDC appointment request is not required for the NDC referral process. 

The rule-based decision logic for the BPA is provided as Appendix A. In summary, the BPA appears during the rooming process at ambulatory office visits for patients with either no information regarding tobacco use in the social history section of the EHR or for patients who have indicated current tobacco use. The BPA does not appear if a NDC referral had already been placed within the past 90 days, or if the patient has indicated that they are a former or never smoker. Additional rule-based restrictions were used within the BPA logic to avoid interference of the BPA with other EHR-related tasks that rooming staff must complete. This approach was chosen to balance staff and patient burden with the goal of continuing to offer interventions at a reasonable interval. 

In conjunction with the BPA, a “SmartPhrase” was designed to facilitate documentation by the oncology provider of conversations related to tobacco use. SmartPhrases are an informatics tool within Epic^©^ that insert pre-populated texts into clinical notes and can be implemented at several levels within the organization. A system-level SmartPhrase can be used by all clinical providers within the organization. The SmartPhrase developed was named “tobacco” and populated the clinical note with the following phrase: “The patient uses tobacco and has been offered an appointment at the Nicotine Dependence Center.” The use of SmartPhrase was optional for the clinicians. 

**Clinical Workflow:** In the new workflow for an ambulatory visit, after a current tobacco user is roomed by the staff and the NDC referral is placed via the BPA, clinicians complete their clinical visit. The patient then “checks out” with scheduling staff to make appointments for further testing or consultative visits. The scheduling staff makes the NDC appointment at the time of check out for patients referred via the BPA. Options for appointments include a face-to-face visit at a centrally-located NDC location (preferred) or a telephone counseling visit.

**Implementation:** The implementation of the informatics processes into clinical practice required the efforts of multiple information technology experts. Reviews by multiple institutional committees were also required, as many aspects of the system were novel to the institution. For example, this BPA was the first to be directed to rooming staff at Mayo Clinic, the first to provide a referral that does not require a provider’s co-signature, and the first to not include a “patient declined” option for referrals. The approvals to allow informatics security access were facilitated by the fact that this was a NCI-supported effort and received strong support from all stakeholders.

The ambulatory practice of the Division of Medical Oncology at Mayo Clinic in Rochester, MN, was selected as the pilot site for the implementation as this practice contacts significant numbers of cancer patients seen at the MCCC. Division leadership was engaged at the onset of implementation and was highly supportive. The rooming staff was also engaged into the workflow development process and contributed significantly to the overall design of the clinical workflow and the BPA. An online education module was developed for training the staff, including the educating of both the new workflow and what services patients received at the NDC. Additionally, an in-person training explaining the new workflow process was conducted for the rooming staff by the NDC staff. 

As part of the overall implementation strategy, patient engagement brochures, provider education brochures and provider engagement posters were developed. A general communications plan publicizing the initiative to the overall institution was designed and implemented by an institutional resource. As physical resources, separate quick reference guides within Epic^©^ were developed for the rooming staff and the providers. All the clinical providers from the Division of Medical Oncology were offered to attend a live presentation from the study investigators and prior to the implementation date; all the clinicians received an email from the Chair of Medical Oncology outlining the new workflow processes in the initiative. 

**Evaluation:** To provide a preliminary estimate of the effectiveness of the referral process via the BPA, the following metrics were evaluated for a 6 week period (15 July 2019 to 23 August 2019) beginning one week after implementation, allowing for a one-week “run in” period. For comparison, the number of patients having an ambulatory visit in the Division of Medical Oncology in the 6 weeks prior to implementation was also determined (27 May 2019 to 5 July 2019). Data sources for the evaluation included analytical reports within Epic**^©^** from the Division of Medical Oncology and the patient appointment reports from the NDC.

## 3. Results

The BPA process was implemented in the Division of Medical Oncology on 8 July 2019. In the 6 weeks prior to the BPA and workflow implementation (27 May 2019 to 5 July 2019), there were 6371 ambulatory visits completed by 4553 unique patients. A total of nine patients from the Division of Medical Oncology had an NDC appointment scheduled. Of these nine patients, four patients completed, two cancelled, and three patients did not attend their appointment. In Epic©, tobacco use status is recorded in the social history section. Prior to the implementation, tobacco use status was missing for nearly 30% of patients in the MCCC cancer patient registry. 

**Figure 2:**
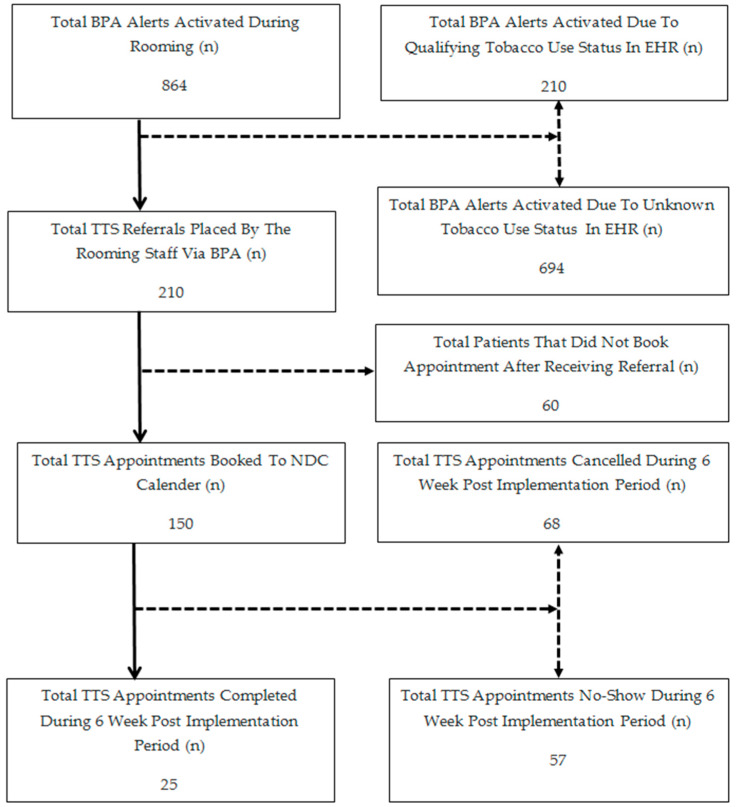
Outcomes of electronic health record (EHR) based best practice advisory (BPA) alerts and tobacco treatment specialist (TTS) appointments for the first six weeks of implementation in the Division of Medical Oncology, Rochester, MN. Values are the number of patients in each category over this period of time. NDC, Nicotine Dependence Center, Rochester, MN.

The post-implementation outcomes in the 6 weeks period (15 July 2019–23 August 2019) are outlined in Figure 2. Over this time, there were 6699 ambulatory visits completed by 4758 unique patients. Of these patients, 864 (18%) received the BPA. Of these 864, 694 (80%) received the BPA because their tobacco use status was unknown (i.e., there was no information available in the tobacco use section of the social history at the time of patient rooming). For the 864 patients receiving the BPA, 654 (76%) were indicated by the room staff to have never used tobacco, have used tobacco > 30 days ago, or were already being treated for tobacco use. All of the remaining 210 patients who received the BPA and were current tobacco users were referred to the NDC by the rooming staff. Of the 210 patients referred to the NDC, 150 (71%) had an NDC appointment scheduled at the completion of their visit with their oncology provider. Of these, 68 (45%) subsequently cancelled their NDC appointment, 57 (38%) did not keep the first scheduled appointment, and 25 (17%) attended their NDC appointment during the 6 week pilot evaluation period (Figure 2). 

## 4. Discussion

The major challenge in helping the cancer patients stop using tobacco is not the lack of efficacious interventions, but that the utilization of these interventions is extremely low in practice. The NCI Cancer Center Cessation Initiative aims to improve the provision of effective tobacco use treatment for all cancer patients.

Attempts to encourage clinicians to provide tobacco use interventions to their patients who use tobacco have had mixed results [16]. For example, approximately 80% of all cigarette smokers in the US see a clinician at least annually [17]. Most (~90%) report that a clinician had asked them about tobacco use during the appointment [18], but only approximately half report being advised to quit using tobacco [19]. Only about 20% receive any form of tobacco cessation counseling during healthcare visits [16] and less than half of these receive pharmacotherapy for tobacco use. The behaviors of oncologists are similar to other clinicians with only about a third providing any assistance to patients according to surveys [20]. Many oncologists cited lack of time, resources, and the expertise to provide effective tobacco use interventions [21] but would welcome the provision of interventions by others [22]. 

There have been several efforts to use EHRs to facilitate the clinician delivery of interventions [23,24,25,26], most of them requiring direct clinician involvement [26,27,28,29,30]. These efforts have included oncology practices [31,32,33]. Although these systems can be efficacious in terms of increasing referral to intervention services, patient utilization of these services, and their overall effectiveness in terms of reducing tobacco use, have been at best modest [34,35,36,37]. Like other physicians, oncologists are also encouraged to help their patients stop using tobacco [38,39,40]. The potential utility of EHR-based clinician reminder and referral systems has been recognized specifically in oncology patients [41]. The initial evaluation of these systems shows encouraging results in terms of reach and acceptability among patients [32,42,43]. These systems require the involvement of oncologists in terms of placing orders for referral and pharmacotherapy [24,31] and have shown promise in terms of screening rates, patient engagement with treatment, and abstinence outcomes [3,31,33]. However, given the previously mentioned barriers to involvement by oncologists and other clinicians, the sustainability of these approaches remains to be determined.

There are two main innovations in our approach. First, we utilized an “opt-out” approach to NDC referral without clinician involvement—all patients who acknowledge current tobacco are referred by default to the NDC, regardless of intent to stop. The main rationale for this recently proposed “opt-out” approach, which has shown promising early results [44], is that the efficacy of tobacco use interventions has little dependence on the intent to stop [45]. Operationally, this approach also has the advantage of not requiring the rooming staff who utilize the BPA to ascertain intent. However, it also required considerable effort to implement. All prior BPAs that included patient referrals had a “patient decline” option; ours was the first to not include this option, which required additional institutional approvals and leadership endorsement. The rationale for this approach was to allow oncology providers to help patients make an informed decision prior to declining the NDC referral provided by the rooming staff; declining the referral is a clinical decision and therefore requires an informed decision-making process with a clinical provider. 

Second, the NDC referral process does not require a proactive step on the part of clinicians. Our formative work confirmed prior findings that clinicians perceive that the clerical burden posed by the EHR can significantly contribute to clinician burnout [46,47] through phenomena such as “alert fatigue” [48]. We thus designed the workflow so that the rooming staff could themselves initiate NDC referral, without the need for a provider to co-sign. This was unprecedented at Mayo Clinic, and required major efforts in terms of securing leadership endorsements, changing the existing institutional practices and informatics optimizations on EHR security access for rooming staff. The institution determined that placing an NDC referral based solely on tobacco use status did not constitute a formal “order”, but was a request for an appointment, and did not require medical decision making since the request was based only on the tobacco use status. Thus, the appointment request did not require a clinician co-sign.

In the initial 6 weeks of implementation, the majority (694/864 (80%)) of BPA alerts were related to the “unknown or absent tobacco use status” within the social history section of the EHR used to document tobacco use status. This proportion is consistent with the preliminary assessment during the BPA design that approximately 30% of cancer patients at our institution had no documentation of tobacco use status. Others have also noted deficiencies in the clinical documentation of tobacco use status in the EHR [49,50] providing an obvious opportunity for practice improvement. Improving the documentation rate would decrease the rate of BPA activation (as the BPA is not activated for never or former tobacco users) and decrease rooming staff workload. In this study, rooming staff were encouraged but not required to manually update the tobacco use status in the EHR. One potential approach to improving documentation would be a change in the current process so that the staff who schedules ambulatory appointments would ascertain current tobacco use status at the time of scheduling. This workflow change, which is currently being explored, would also subsequently improve the unnecessary firing for the BPA due to the lack of information in file. Meanwhile, patients who received the BPA alert due to unknown or absent tobacco use status in the EHR will continue to get a BPA alert every 90 days until the tobacco use status is manually updated by a clinical provider. 

Patients qualified to receive an NDC referral were in fact referred during the rooming process, indicating the excellent compliance of the rooming staff with the new process. Anecdotally, the feedback obtained from the rooming staff through meetings and one-on-one conversations was uniformly positive. In particular, the rooming staff commented that they appreciated the opportunity to directly contribute to an important aspect of patient care. 

Although approximately two-thirds of the patients referred to the NDC during the rooming process had an appointment scheduled at the time of check-out, less than two of 10 patients who received referrals completed an NDC appointment within the first 6 weeks of implementation. This represented a substantial increase in the number of completed appointments compared with the 6 weeks before the pilot implementation period (25 completions vs. four completions), but represents an opportunity for improvement. Other studies also suggest that one of the major challenges to improving tobacco treatment among cancer patients has been the low rate of participation by the patients [6,51,52,53,54]. 

Future work will be necessary to determine how best to address the barriers to participation and increase the rate at which patients who are referred to the NDC attend their appointments [55]. For example, rooming staff noted that some patients may be concerned about the cost of a NDC appointment. Although the Affordable Care Act requires that tobacco cessation counseling and medications are covered at no cost to the patient, coverage varies by employer or plan. All plans in Minnesota’s health insurance marketplace require coverage of tobacco cessation counseling and medications with no cost sharing, but efforts may be needed to educate both patients and providers about this feature. Although the increase in patients attending NDC appointments in the first 6 weeks of implementation was encouraging, the sustainability of the initial implementation will also need to be evaluated in future work.

## 5. Conclusions

An EHR-based “opt-out” approach to refer cancer patients to tobacco use treatment that does not require active involvement by clinical providers is feasible within oncology clinical practices. Preliminary experience suggests that the majority of cancer patients who use tobacco are scheduled for treatment, but that further work is needed to increase the proportion of scheduled patients who attend their appointments. This EHR-based “opt-out” approach could also be applied for other types of patients in other ambulatory practices, further expanding the reach of tobacco use treatment.

## Appendix A

Clinical informatics logics used to create the best practice advisory (BPA).

**Base Records**: BPA alerts for patients that are current tobacco, smokeless tobacco or e-cigarette users that have not had a nicotine dependence counseling order placed in the last 90 days. The user is directed to place nicotine dependence consult order.

**Criteria Records:**

1. Current Smoking Tobacco Use = Current every day OR Current some day OR Light tobacco smoker OR Heavy tobacco smoker OR Unknown tobacco use status OR Smoker but current status unknown OR No information on file
2. Current Smokeless Tobacco Use = Current User
3. Current E-cigarette Use = Current Every Day User OR Current Some Day User
4. Nicotine Dependence Consult Referral active OR completed in the last 90 days.
5. Nicotine Dependence Treatment Episode Of Care is active in file
6. Nicotine Dependence Consult Referral is pended in file
7. FYI flags “Hospice” OR “Hospice-External” in file
8. Patient has an active status in “Nursing Home Patient” registry in file
9. Patient “Has Legal Guardian” flag in file

**Decision Logic**: (At least 1 of (1:3) AND exactly 0 of (4:9)) + Restrictions *

* **Department Inclusion Restrictions**: Department codes where the BPA workflow is implemented.

* **Provider Type Inclusion Restrictions**: Clinical Assistant, Clerk, Patient Care Assistant, Technician Unlicensed

* **Encounter Type Exclusions Restrictions**: Clinical Communication, Refill, Letter, Patient Message, Transcribe Orders, Outside Material Tracking, Reconcile Outside Data, Confidential, Procedure Visit, Telemedicine, Prep for Case, Lab, Treatment, Infusion, E-Review, E-Tumor Board, External Outreach, Internal E-Consult, External E-Consult, Nurse Only, E-Consult, Ancillary Procedure, Historical Appointment, Clinical Support, Abstract, Ancillary Orders, Anticoagulation Visit, Charge Only, Documentation, Education, Emergency Response, Episode Changes, Hospice F2F Visit, Lactation Consult, Lactation Encounter, Medication Management, Orders Only, Patient Outreach, Remote Monitoring, Specialty Pharmacy, Suspected Abuse, Telephone.
